# Spatial population dynamics and temporal analysis of the distribution of *Lutzomyia longipalpis* (Lutz & Neiva, 1912) (Diptera: Psychodidae: Phlebotominae) in the city of Clorinda, Formosa, Argentina

**DOI:** 10.1186/s13071-017-2296-0

**Published:** 2017-07-25

**Authors:** Andrea Gómez-Bravo, Alba German, Marcelo Abril, Marcelo Scavuzzo, Oscar D. Salomón

**Affiliations:** 1 Fundación Mundo Sano, Paraguay 1535, Ciudad Autónoma de Buenos Aires, Argentina; 2 Instituto de Altos Estudios Espaciales “Mario Gulich”, Centro Espacial Teófilo Tabanera, Ruta Provincial C45 a 8 Km, Falda de Cañete, Córdoba, Argentina; 3 Instituto Nacional de Medicina Tropical, Neuquén y Jujuy, Puerto Iguazú, Argentina; 4 Consejo Nacional de Investigaciones Científicas y Técnicas, Godoy Cruz 2290, Ciudad Autónoma de Buenos Aires, Argentina

**Keywords:** *Lutzomyia Longipalpis*, Phlebotominae, Visceral leishmaniasis, Environmental variables, Spatio-temporal model

## Abstract

**Background:**

*Lutzomyia longipalpis*, the vector for the causal agent of visceral leishmaniasis (VL), has extended its distribution in the southern cone in the Americas. The first urban record of *Lu. longipalpis* in Argentina was from the City of Clorinda in 2004. The aim of this study was to analyse the monthly distribution and abundance of *Lu. longipalpis* and to evaluate its association with environmental and climatic variables in Clorinda City, Province of Formosa.

**Methods:**

Phlebotominae sampling was performed using CDC light mini-traps that were placed in different sites of the city between January 2012 and December 2013. Environmental variables including the normalised difference vegetation index, normalized difference water index, land surface temperature and precipitation were evaluated using a spatiotemporal model.

**Results:**

A total of 4996 phlebotomine sandflies were captured during the study period, and eight species were reported: *Lu. longipalpis*, *Migonemyia migonei*, *Nyssomyia whitmani*, *Ny. neivai*, *Brumptomyia guimaraesi*, *Evandromyia cortelezzii/sallesi*, *Psathyromyia bigeniculata* and *Expapillata firmatoi*. This is the first urban record of *Ex. firmatoi* in Argentina. *Lutzomyia longipalpis* was the most abundant species between 2012 and 2013, and it appeared in all the sampled sites. Moreover, the model applied showed that ground humidity and temperature were significantly associated with the abundance of *Lu. longipalpis*.

**Conclusions:**

This longitudinal approach at city scale allows for modelling that explains more than 60% of the temporal variability of the abundance of *Lu*. *longipalpis* based exclusively on satellite obtained data. The results support the hypothesis of steady ‘hot spots’ of abundance with time, while other sites could change its abundance due to eventual microenvironment changes. The *Lu*. *longipalpis* abundance driving factors are breeding site-related variables, highlighting the importance both for modelling and surveillance to use lag data.

## Background

Neglected diseases are a group of infectious diseases, usually parasitic and chronic, that principally affect poor populations with limited access to healthcare. The World Health Organization (WHO) has listed 17 diseases within this group and among them is leishmaniasis [1]. Leishmaniasis is a group of zoonotic diseases caused by more than 20 species of parasites of the genus *Leishmania* (Trypanosomatidae) and is transmitted to humans through the bite of a hematophagous insect belonging to the family Psychodidae, subfamily Phlebotominae. There are two main clinical manifestations of leishmaniasis caused by these parasites: tegumentary (cutaneous and mucocutaneous) and visceral or kala-azar (VL). These types not only vary clinically but also on the vector, the reservoir and the species of parasite involved.

Of these two, VL is the most serious, with an estimated worldwide incidence of 200,000 to 400,000 cases per year [2]. American VL, caused by *Leishmania infantum*, has a case-fatality rate of 6.6% and Brazil reports more than 96% of the cases, but the Southern Cone (South-Midwest of Brazil, Paraguay and Argentina) showed the most significant increase in VL incidence in the Americas during this century [3]. This is associated with the southern spread of *L. infantum* and its main vector, the synanthropic phlebotomine *Lutzomyia longipalpis* which led to a new scenario of transmission in urban areas [4, 5].

In the South-Midwest of Brazil, in Mato Grosso do Sul State, the epidemic pattern related to anthropogenic environmental disturbances and migration generated a human VL report increase of 1510% between 2001 and 2006 in Campo Grande City [6, 7]. The highest climatic suitability for *Lu. longipalpis* has been observed in the southern area of the state on the border with Paraguay, including Ponta Porá and even in northern Paraguay [8, 9]. Consequently, Paraguay reported one human VL case during the year 2000, but afterward an average of 111 cases/year (75–145) between the years 2010 and 2015; 90% of these are from the most populated area (Asunción Capital District, and departments of Central, Paraguarí and Cordillera) [10, 11]. However, the canine VL seroprevalence in Asuncion City already ranged from 3.1 to 11.8% between 1997 and 1999 [12], and it reached up to 48.9% in Lambare (Asuncion Capital District) in a survey performed in 2004–2006 [13].

In 2004, *Lu. longipalpis* associated with urban scenarios in Argentina was first reported [14]. After this report from Clorinda City and-Puerto Pilcomayo, new reports followed, providing evidence for the spread of *Lu. longipalpis*. The first human VL autochthonous case in Posadas occurred in 2006, 295 km east from Clorinda also bordering with Paraguay [15]. In 2010, *Lu. longipalpis* was first recorded in Salto, Uruguay, 675 km south from Clorinda bordering with Argentina [16]. In 2013, *Lu. longipalpis* was first recorded in Tartagal, Argentina, 692 km north-west from Clorinda and 60 km from the border with Bolivia [17].

The first urban captures of *Lu. longipalpis* in Argentina during June and December 2004 were in three out of 18 sampled sites, one in a courtyard within the city of Clorinda and two in the contiguous rural-periurban Puerto Pilcomayo associated with pigsties [14]. In surveys performed during October 2007 in the same area, *Lu. longipalpis* was present in 10 out of 140 sampled sites again in Clorinda-Puerto Pilcomayo, including those sites were vector presence was observed in 2004 [18]. Despite the fact that the vector found in Asuncion belongs to the pheromone *Lu. longipalpis* species complex associated with urban spread [19, 20], in Clorinda the results after 3 years of the first report showed a clustered pattern with restricted dispersion. Clorinda is 40 km away from Downtown Asuncion, and there is an intense human transit through bridges for pedestrians and automobiles that connect both cities.

Given this situation, this study was conducted 5 years after the second survey in both Clorinda and Puerto Pilcomayo to assess the monthly distribution and abundance of *Lu. longipalpis* in sites with previous captures and sites with no previous captures, and to evaluate its association with environmental and climatic satellite variables. The results presented here aim to provide an insight into the spatial population dynamics at the city spatial-scale. We hope these results contribute to the understanding of the permanence-expansion of VL vectors in urban areas in the Americas and facilitate the design of improved time-space focused surveillance and control strategies at the mesoscale/urban focus level.

## Methods

### Study area

**Fig. 1:**
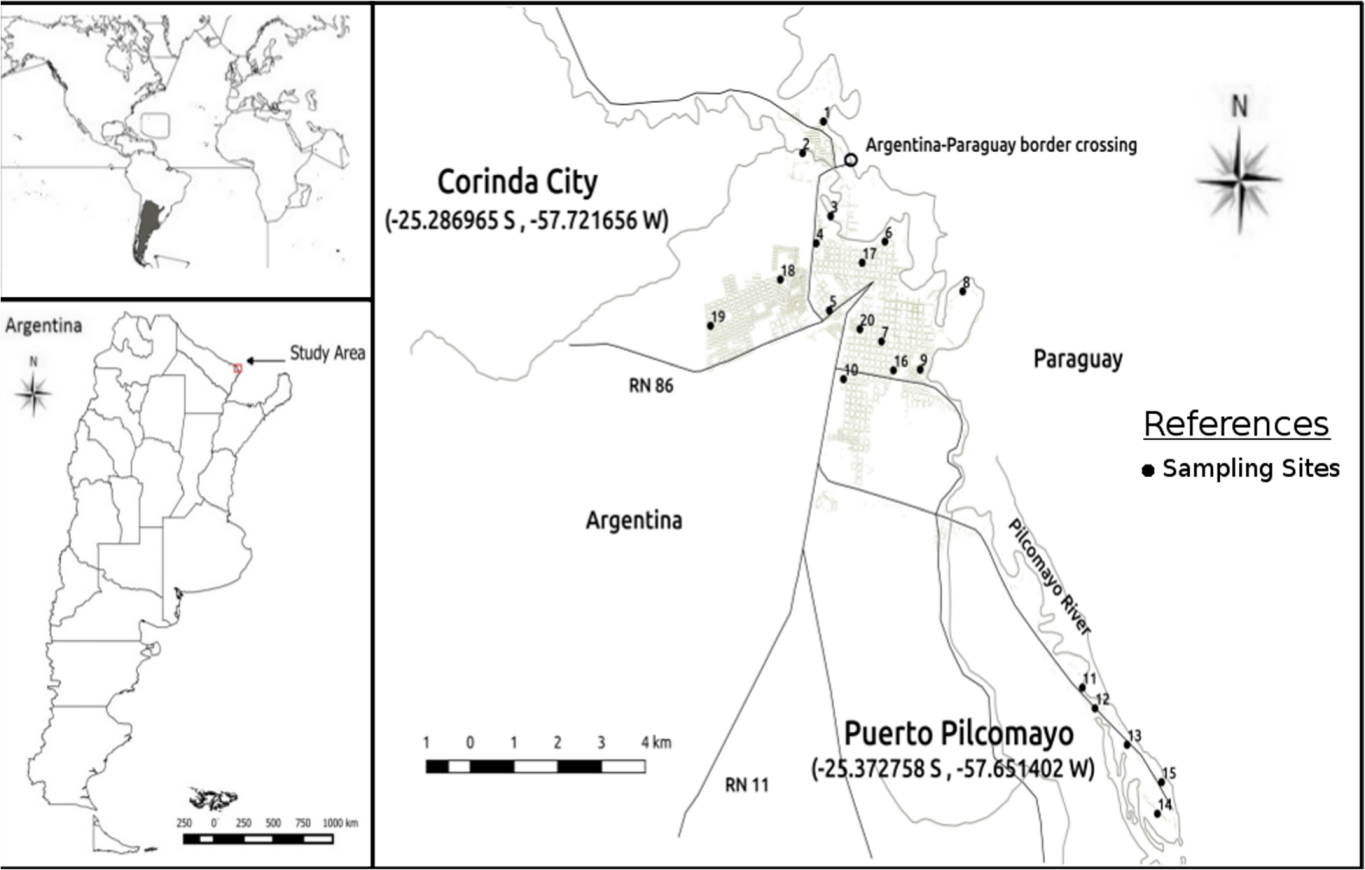
Study area, Clorinda-Puerto Pilcomayo, Argentina. The numbered points make reference to the location of the sampling sites

Clorinda City (25°17′S, 57°43′W, and 62 m above sea level) is located in Formosa Province, on the Pilcomayo-Paraguay River which is also the international border between Argentina and Paraguay (Fig. 1). Three pedestrian bridges and many fords link Clorinda to Nananawa in Paraguay, which is 40 km from Asunción, Paraguay’s capital city and 115 km from the capital of the Province of Formosa. The city belongs to the Eastern (humid) Chaco ecoregion [21]. The climate is subtropical without a dry season, humid subtropical climate (Cfa) type according to the Köeppen-Greiger classification, with a mean annual median temperature of 23 °C (ranging from -2 °C to 43 °C), and an average annual rainfall of 1300 mm. The driest period in this region is from June to August [22]. Clorinda’s urban area has 53,506 inhabitants distributed in 980 blocks, while Puerto Pilcomayo is an area of 669 inhabitants contiguous to the south-east border of the city where rural-periurban houses are scattered along 11 km on the river bank [23].

### Phlebotominae sampling

CDC light mini-traps [24] were placed in peridomestic habitats overnight during two consecutive nights once a month between January 2012 and December 2013. The captures were performed during the third week of the month, but if the rain prevented the activity, the sampling was performed during the fourth week of the month. This only happened twice, during February 2012 and June 2013. The sample sites were located mostly in Clorinda City (15) and five were located in Puerto Pilcomayo, including the ten sites with previous records of *Lu. longipalpis* [14, 21]. Five sites within Clorinda with no previous records of the vector but with environments prone to have phlebotomine [25] were also included. Species identification was performed following Galati’s key [26] with abbreviations according to Marcondes [27].

### Environmental variables and correlation analyses

The normalised difference vegetation index (NDVI), and normalised difference water index (NDWI) from MODIS MOD13Q1 satellite products with capture every 16 days at 250-m spatial resolution were used [28–30]. NDVI and NDWI are dimensionless indexes that range between -1 and 1. For NDVI, negatives value mean water, values near to 0 mean bare soil and for positive values, the NDVI grow when there is more abundant and denser vegetation. Land surface temperature (LST) from MODIS MOD11A2 satellite product, stored on a 1-km Sinusoidal grid is the average value of clear-sky LSTs during an 8-day period, validated at Stage-2, corresponding to daytime (LSTday) and to night time (LSTnight) as maximum and minimum temperature [31]. Local precipitation was obtained from the tropical rainfall measuring mission (TRMM) [32].

To explore the association of these variables with *Lu. longipalpis* abundance, the following were computed: (i) time series and mean monthly values for each variable; (ii) mean values of all the pixels involved according to the satellite product pixel area for each site; (iii) time lags of these mean values with one (lag1), two (lag2) and three (lag3) months before the sampling month; (iv) the Pearson correlation between variables at each time; (v) a generalized linear model (GLM), with a Poisson *Lu. longipalpis* abundance distribution, to explain vector abundance with the variables that show the highest correlation and significance values [33, 34].

## Results

In the two-year trapping period, 4996 Phlebotomine were captured belonging to eight species. In 2012, *Lu. longipalpis* was the most prevalent with 98.53% of the total captures. In 2013, *Lu. longipalpis* was also the most prevalent, but the captures were lower than in 2012 (89.13%), while *Mg. migonei*, *Nyssomyia whitmani*, *Ny. neivai* and *Evandromyia cortellezzii/sallesi* captures were higher, accounting for 20.89% of the entire phlebotomine population in 2013. Furthermore, two species, *Psathyromyia bigeniculata* and *Expapillata firmatoi*, that were not found in 2012 were captured in 2013 (Table 1).

**Table 1:** Phlebotomines captured and percent (%) by year and species, from January 2012 to December 2013, Clorinda-Puerto Pilcomayo, Argentina

Species	Year 2012		Year 2013		Total	
	*n*	%	*n*	%	*n*	%
*Lutzomyia longipalpis*	2540	98.53	1913	79.11	4453	89.13
*Migonemyia migonei*	13	0.5	182	7.53	195	3.90
*Nyssomyia whitmani*	12	0.47	156	6.45	168	3.36
*Brumptomyia guimaraesi*	2	0.08	1	0.04	3	0.06
*Nyssomyia neivai*	2	0.08	90	3.72	92	1.84
*Evandromyia cortelezzii/sallesi*	9	0.35	70	2.89	79	1.58
*Expapillata firmatoi*	0	0	4	0.17	4	0.08
*Psathyromyia bigeniculata*	0	0	2	0.08	2	0.04
Total	2578	100	2418	100	4996	100

**Fig. 2:**
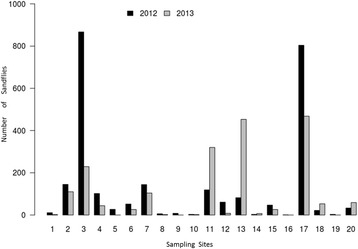
*Lutzomyia longipalpis* cumulative abundance by year and sampling site, Clorinda-Puerto Pilcomayo, Argentina

*Lutzomyia longipalpis* appeared in all the sampled sites, but there were differences in abundance among sites and years (Fig. 2). Sites 3 and 17 showed the highest abundances over the capture period. In 2013, there was a noteworthy increase of abundance in sites 11 and 13 located in Puerto Pilcomayo, while most of the other sites had less *Lu. longipalpis* during the second year (Fig. 2).

**Fig. 3:**
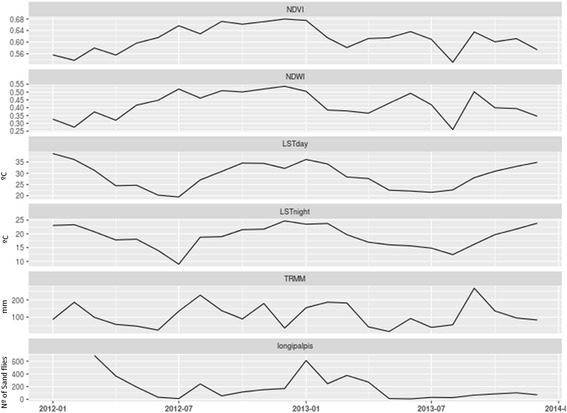
Twenty-four months temporal series of NDVI, NDWI, LSTday, LSTnight, TRMM (rain) and *Lu. longipalpis* abundance

The monthly time series of environmental variables obtained is presented in Fig. 3 jointly to *Lu. longipalpis* abundance. Here it is possible to observe the different temporal patterns of the environmental variables and the correlation between them. While the peak of abundance was coincidental with high values of almost all variables except TRMM, other periods of increase in the variables values are not associated with higher abundances of *Lu. longipalpis*. The temporal pattern of abundance of *Lu. longipalpis* seems not to follow the pattern of just one critical variable of this environmental series through the studied period. This fact shows that it is a complex system where many variables influenced together on several scales, even some not included in this macro-environmental variables analysis.

**Fig. 4:**
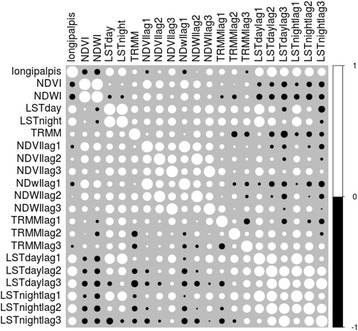
Pearson correlation between environmental variables and *Lu. longipalpis* abundance. Colour and shape of the circles are proportional to the correlation coefficients. *Abbreviations*: NDVI: normalised difference vegetation index, NDWI: normalised difference water index, LST: land surface temperature, TRMM: tropical rainfall measuring mission

However, to better assess the relationship between *Lu. longipalpis* abundance and the environmental variables, a correlation analysis was made adding the three lag functions calculated. The results are shown in Fig. 4. To measure the importance and significance of the Pearson correlations, the *P*-values were also analysed (Table 2). The only significant correlation between the environmental and climatic variables and *Lu. longipalpis* abundance during the capture period was the dependent variable LST and LST time lags.

**Table 2:** Pearson correlation and *P*-value between the environmental variables and *Lu. longipalpis* abundance

	*Lu. longipalpis*
NDVI	-0.194
NDWI	-0.239
LSTday	0.493*
LSTnight	0.489*
TRMM	0.144
NDVIlag1	-0.077
NDVIlag2	0.056
NDVIllag3	0.347
NDwIlag1	-0.121
NDWIlag2	0.008
NDWIlag3	0.318
TRMMlag1	0.244
TRMMlag2	0.208
TRMMlag3	-0.047
LSTdaylag1	0.555**
LSTdaylag2	0.720**
LSTdaylag3	0.554**
LSTnightlag1	0.580**
LSTnightlag2	0.587**
LSTnightlag3	0.467*

*P < 0.05

**P < 0.01

With these results, a generalised linear model was tested (Table 3). In the formula, each environmental variable was included, using the most strongly correlated function. LSTdaylag2, LSTnightlag2, NDVIlag3, NDWIlag3, TRMMlag1, were used to represent LST, NDVI, NDWI and TRMM, respectively. The family used in the model was Poisson, representing the distribution of the abundance variable.

**Table 3:** Results of the generalised linear model

	Estimate	Std. Error	*Z*-value	Pr(>|z|)	Significance
Intercept	2.61E + 000	6.61E-001	3.944	8.03e^-5^	***
LSTnightlag	-2.31E-002	1.27E-002	-1.82	0.06881	
LSTdaylag2	1.43E-001	8.75E-003	16.346	< 2e^-16^	***
NDVIlag3	-5.28E + 000	8.75E-003	-2.985	0.00283	**
TRMMlag1	1.72E-004	3.25E-004	0.528	0.5972	
NDWIlag3	4.295	9.57E-001	4.486	7.27e^-6^	***

**P < 0.05

***P < 0.01

**Fig. 5:**
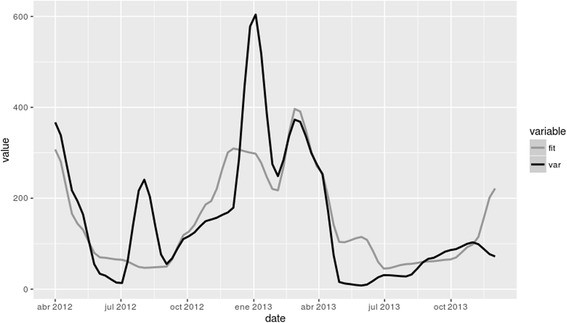
Abundance of *Lu. longipalpis* during the study period, both in situ (*black line*) and modelled (*grey line*)

The actual abundance of *Lu. longipalpis* obtained through captures and the simulated abundance generate had a good correlation (Pearson coefficient 0.75, *P*-value = 1e^-04^). The total and simulated abundances are plotted in Fig. 5.

## Discussion

Satellite information of environmental variables was used to analyse the influence of these on the abundance of *Lu. longipalpis*. This is considered a robust input to explain vectors abundance [29, 35, 36]. This methodology has been tested for other vectors such as *Aedes aegypti* [22, 37] but in this study, we developed a specific methodology for *Lu. longipalpis* that included more variables and the use of a different statistical approach. This methodology is more robust than in previous studies of vector abundance due to the statistical nature of the model used (GLM), which has been tested to be better suited for analysing ecological relationships, which can be poorly represented by classical Gaussian distributions [34, 38].

Even though *Lu. longipalpis* was first reported in Argentina in 1951 [39], it was not until 2004 that Argentina was considered at risk for VL transmission given that the vector was found for the first time in an urban setting (i.e. in Clorinda) [14]. Although the first human case of VL in Argentina occurred in 1924 [40], the first confirmed autochthonous case in the country was not reported until 2006, with a human case caused by vectorial transmission of the parasite from an infected canine [15]. This was after Brazil, and Paraguay first reported cases of VL in humans in Mato Grosso do Sul in 1998 and Asunción in the year 2000 [41–43]. Currently, VL is autochthonous in 12 countries of the Americas; between 2001 and 2013, 45,490 cases were registered with an annual average of 3499 cases [3].

This study was performed in the city of Clorinda 11 years after the first report of *Lu. longipalpis*. A total of eight phlebotomine species were registered, including *Lu. longipalpis, Mg. migonei, Br. guimaraesi*, *Ny. neivai*, *Evandromyia cortellezzii/sallesi*, *Ny. whitmani*, *Ex. firmatoi* and *Pa. bigeniculata*; the last three species had not been previously reported in other studies from Clorinda [18]. Moreover, this is the first time that *Ex. firmatoi* is reported in an urban scenario in Argentina.

*Lutzomyia longipalpis* was the most abundant species, representing 89.13% of the total specimens captured. This is also the highest percent abundance registered since 2009 when *Lu. longipalpis* was found in ten areas of Clorinda and Puerto Pilcomayo where it represented 80.6% of the captured phlebotomine, with a male prevalence of 75.9% [18].

These results confirm the urbanisation trend of *Lu*. *longipalpis* and visceral leishmaniasis urban transmission in the southern cone of the Americas, in areas with previous records of cutaneous leishmaniasis, mainly due to rural and periurban transmission. Regular entomological surveillance, together with VL case surveillance via increased awareness of human and domestic animals health agents, should be performed in vulnerable areas without reports of *Lu. longipalpis* or cases.

Throughout the study, variations were found regarding the species frequency and the representability of the species captured. Nonetheless, *Lu. longipalpis* was the species with the highest representation, with 98.5 and 74.1% of the total of species captured between 2012 and 2013. These results are similar to those reported by other authors in studies performed in urban scenarios from other areas of northern Argentina like Posadas, Misiones [25, 44], Santo Tome, Corrientes [45], Tartagal, Salta [17] and Mato Grosso do Sul, Brazil [46].

Regarding the periods of highest abundance, summer and the beginning of autumn were the times of the year with the greatest quantity of phlebotomine captured, while winter was the time with the least number of them. These results are in agreement with studies performed by de Oliveira et al. [41] where the greatest quantity of captured phlebotomine was during the month of February after a period of abundant rain.

The capture sites covered different environments present in the urban areas of Clorinda and rural areas of Puerto Pilcomayo. Nonetheless, not all the sites showed the same abundance of *Lu. longipalpis*. In 2012, sites 3 and 17 were the sites with the highest abundance, but in the same period in 2013, the sites with the highest abundance where sites 11, 13 and 17. A study performed by Fernandez et al. [47] with a time-transversal sampling strategy indicated that the distribution of *Lu. longipalpis* in Posadas followed a time-variable “island” pattern of high abundance or ‘hot spots’. The current study conducted in Clorinda during 2 years of sampling has shown is that some of these “islands” are stable and others are variable through time.

With the constant/continuous sampling data obtained in this study, it was possible to determine a model to evaluate environmental variables that might influence the population dynamics of *Lu. longipalpis*. This is not always considered due to the sampling time-transversal methodology constraints seen in most studies. The model showed that ground humidity and temperature are the variables that could be significantly determining the abundance of the species and also but not equally strong, the vegetation in the area. Land surface temperature (LST), in particular, is a key variable in climatological and environmental studies [31, 48–52] and the current study has shown a high, positive and significant correlation with the abundance of *Lu. longipalpis.*

The variables represented by NDVI and NDWI could be eventually associated with the abundance of phlebotomines through the characterisation of breeding sites. Optimal breeding sites have a high content of organic matter in decomposition, soil availability and humidity but scarce vegetation and usually have some domestic or peridomestic use. Examples of breeding sites include, but are not limited to, abandoned buildings, the soil in human dwellings, animal burrows, street rubbish, soil at the base of old walls, dry excreta from small domestic animals and soil cracks [53].

More specifically, studies on breeding sites of *Lu. longipalpis* from Brazil [4, 53] have shown that the sites with the greatest rates of abundance were animal pens and between rocks. Both of these types of sites have very low values of NDVI, which measures the vegetation cover of a surface, referring to the amount of chlorophyll, which may explain the negative association seen in the current model. On the other hand, these same authors characterise breeding sites as places with humidity, which is in agreement with the positive correlation found for NDWI in the current study.

It is important to note that in the present work we have been able to obtain a model that represents more than 60% of the temporal variability of abundance based exclusively on satellite variables. Given that *Lu. longipalpis* is a species with a high dependency on microscale factors, a value of 60% is satisfactory for our purposes and it would be hard to obtain a value much higher than the one obtained herein. However, a negative point of the model is that it does not generate a good representation of some peaks of abundance (Jul 2012 Jan 2013). In order to be able to elucidate this difference, it would be necessary to perform further analyses. Regardless, it is worth higlighting that this model uses delayed satellite variables (from 1 or 2 months ago), which means that it is an excellent tool for temporary prediction and can therefore be used for the planning of control actions by the health authorities.

## Conclusions

This study represents a macro spatial approach of the VL vector in the city, focused on its temporal distribution, different from other approaches [25, 54]. The results of this study highlight the importance of designing and applying tailored strategies for entomological surveillance control that are based on the different epidemiological scenarios and population dynamics of *Lu*. *longipalpis*. These results support the hypothesis of ‘hot spots’ of abundance through time that could be forecasted by this remote sensing approach, while other sites increase or decrease its abundance from year to year due to microenvironmental changes that require micro-scale analysis, which is already being tested in ongoing studies. Abundance of *Lu*. *longipalpis* throughout the year was driven by breeding site-related variables at the city scale, such as surface temperature and ground humidity, highlighting the importance of using lag data, both for modelling and surveillance, which once validated will provide enough time to perform a preventive intervention in the sites with higher abundances.

## Abbreviations

GLM: Generalised linear model
LST: Land surface temperature
NDVI: Normalised difference vegetation index
NDWI: Normalised difference water index
TRMM: Tropical rainfall measuring mission
VL: Visceral leishmaniasis
WHO: World Health Organization
